# Classification of Surface Vehicle Propeller Cavitation Noise Using Spectrogram Processing in Combination with Convolution Neural Network

**DOI:** 10.3390/s21103353

**Published:** 2021-05-12

**Authors:** Nhat Hoang Bach, Le Ha Vu, Van Duc Nguyen

**Affiliations:** 1 Institute of Electronics, Hoang Sam Street, Hanoi 10000, Vietnam; hoangbach2509@gmail.com (N.H.B.); vuleha.vdt@gmail.com (L.H.V.); 2 School of Electronics and Telecommunications, Hanoi University of Science and Technology, Dai Co Viet Street, Hanoi 10000, Vietnam

**Keywords:** passive sonar, short time Fourier transform, convolution neural network

## Abstract

This paper proposes a method to enhance the quality of detecting and classifying surface vehicle propeller cavitation noise (VPCN) in shallow water by using the improved Detection Envelope Modulation On Noise (DEMON) algorithm in combination with the modified Convolution Neural Network (CNN). To improve the quality of the VPCN spectrogram signal, we apply the DEMON algorithm while analyzing the amplitude variation (AV) to detect the fundamental frequencies of the VPCN signal. To enhance the performance of the traditional CNN, we adapt the size of the sliding window in accordance with the properties of the VPCN spectrogram data, and also reconstruct the CNN layer structure. As for the results, the fundamental frequencies contented in the VPCN spectrogram data can be detected. The analytical results based on the measured data show that the accuracy of the VPCN classification obtained by the proposed method is above 90%, which is higher than those obtained by traditional methods.

## 1. Introduction

Over the past few years, the progress of science and technology has helped people further explore the ocean by using advanced equipment. However, all radio frequency-based devices lose their effect on targets submerged in deep water. In such conditions, replacing radio waves with sound navigation and ranging (Sonar) is the most optimal solution [1]. The Sonar system is used to perform different tasks, such as observation, detection, navigation, control, and communication [2]. The requirement for real-time detection and classification of underwater signals is of utmost importance. Passive sonar is mainly used to detect noise from marine objects, such as submarines, ships, and marine animals [3]. It does not emit any signals; instead, it only detects sound waves coming to itself. In particular, the processing of passive sonar signals poses a complicated problem due to the changes in time and spectral characteristics of signals even obtained from the same source. According to Nielsen [4], Urich [5], and Brekhovskikh [6], when a ship moves, it generates its own signature signals:

- From generation by engines, machines and equipment onboard while in motion.
- From hydrodynamic flow on the hull.
- From presser foot and motor noise.

Each type of signal has its own characteristics and can be detected by experienced surveyors by hearing or seeing the signal spectrum. Even though these signals are often considered to be noise for telecommunication systems due to their negative impacts on transmission, they are extremely useful for passive sonar, because they carry the full characteristics of the target. During movement, the main noise source of each ship is the cavitation of the propeller blades (accounted for about 80–85% of the noise intensity generated in the marine environment) [7]. The characteristics of this noise depend on the rotation frequency of the propeller blades. The cavitation noise increases proportionately with the speed of the blades and decreases as the depth increases. In the case of low-speed surface ships, noise is mainly caused by engines (main propulsion or diesel generators). The maximum value of the interference spectral density is calculated to be roughly 140 dB ref.1 µPa@1m for small fishing vessels and roughly 195 dB ref.1 µPa@1m for ocean cargo ships [4,6]. For large vessels, there have already been specific models and formulae to estimate the maximum noise level that they emit [6]. However, for small, fast-moving ships (especially divers), we cannot determine their existence by these models; rather, we need to use other methods. The essence of the passive sonar is to process the affected cyclic signals against a very loud background. This re-circulation is caused by the rotation of the propeller blades or the motor. A simple way to separate these cyclic signals from the background is to use the synchronous mediation method. This mediation will remove all components except for the cyclic component, because the mediation part has equal magnitude compared to the period of the cyclic part. This method is very useful in theoretical calculations, but it is difficult to apply in practice, because it is very difficult to accurately identify the period of the cyclic component and the initial phase of the signals.

To overcome these shortcomings, Detection Envelope Modulation On Noise (DEMON) algorithms have been used [4]. DEMON-type algorithms, first introduced by Nielsen in 1991, are an analysis of propeller blade properties, such as the number of rotating shafts, the rotation frequency of the shafts, and the rotation speed of the propeller blades. Since then, there have been many variations of the DEMON algorithm proposed to solve different specific problems, such as the tracking of multiple sources in a decoupled way [8], and 3/2D spectral analysis to extract propeller features from acoustic vector sensor data [9]. The basic DEMON algorithm has been tested in practice [10] and has also been used to detect the breathing pattern of divers from recorded data [11]. These above analyses are based on spectral estimation to detect and classify targets. The detection is performed using classical signal demodulation to obtain the propeller blade characteristics. Additionally, the noise emitted from the targets may vary depending on operating conditions, which affects the stability of the passive sonar signal. Therefore, the real-time statistical changes of those signals must always be monitored. When a change is detected, the processing phase must be rebuilt to re-calculate the results. Research by Yang in 2020 [12] demonstrated that classifications using neural networks often produce high levels of accuracy.

In Industry 4.0, using and mastering AI hardware and software technologies is extremely important, as the applications of AI make data-processing more accurate. The advances and potentials of Machine Learning (ML), including Deep Learning (DL) in the field of acoustics in general and particularly ocean acoustics increasingly attract awareness. Machine learning is a group of specialized techniques that use mathematical and statistical calculations to automatically detect patterns in data. Based on continuously trained data, ML analysis results in the complex relationship between observed data and the desired label; the larger the amount of training data which can be produced, the more accurate specification model ML can give. For passive sonar, Support Vector Machines (SVM) and the ML algorithm are applied to process signals coming from classes of which properties are unknown. In other words, those unknown properties have not been included in the existing database. The basic approach is “one-class” classifications, in which all data from already known classes are considered a single class. According to [13], using the results of the analysis of acoustic signals on the time and frequency domains combined with Least Mean Square as input for the SVM model gives significantly improved results compared to comparable models. Yang’s research team [14] (2018) used unsorted SONAR data for pre-training to increase the effectiveness of supervised learning. They used the values of the final hidden layer of the competitive deep-belief network (CDBF) as the input for the SVM classifier. In 2018, Ke et al. [15] also used multi-layer auto-encoders to pre-train data, where characteristics for noise source classification were used as SVM input in the classification step. However, there are still limitations in classification results, mainly due to two reasons: First, because the complicated underwater environment contains a variety of background noises, and the quality of the received signal is very poor, resulting in a low classification rate; second, because traditional machine-learning methods often extract features manually, which significantly reduces the quality of the training set.

More modern approaches are used for new detection techniques, such as Artificial Neural Networks (ANN), Deep Learning, and so forth. Passive sonar data are passed through algorithms to reduce the data dimension. The now more simplified data will be used in ANN models to detect new patterns, and a threshold will be set to decide if the next event belongs to the new class or not. Here, the detection principle compares known data and received data. In recent years, DL model has had the ability to go into and process hidden features of the target signal through a multi-layer network without mimicking features. Compared with ML methods, DL can extract more specific features of a target through a multi-layer network with greater accuracy. From the proposal of Fukushima (1980) [16] and LeCun (1989), the CNN completed in 2012 [17] was the first multi-layer structure that used relative relationships in space to reduce the parameter dimensions and improve training performance. VGG-16 [18] (2014) formed a trend to improve the accuracy of DL networks by increasing the depth of the model. Variations of GoogleNet [19] (2015), by combining multiple filters of different sizes into the same block, produced the block architecture for the later CNN. ResNet-50 [20] (2016) used identified “short-cut” connections to map inputs from the previous layers to the following layers. It is very deep-network architecture, but has a smaller number of parameters, based on techniques from GoogleNet. DenseNet [21] (2017) is the next generation of ResNet which inherits the block architecture and develops the “short-cut” connection for a dense network. Deep learning can be used to solve the problems of low recognition accuracy when the signal is unstable, as it can extract features based on large datasets. On the other hand, since there is a variety of noise, a good selection of features can improve model performance.

From the above analyses, this research proposed a pre-processing model using improved DEMON for better input to a customized CNN to enhance classification accuracy. The next parts of the paper will be organized as follows: Section 2 introduces the underwater signal pre-processing method, Section 3 analyzes the CNN proposed structure, Section 4 provides the experiment results discussion, and Section 5 concludes the paper. The last part will be our acknowledgement.

## 2. Pre-Processing Underwater Signal

The signals generated from moving ships can be divided into two main types of components: speed-dependent and non-speed-dependent. The speed-dependent components are the negative signals generated by the ship’s propulsion systems (engine, gearbox, rotating shaft, propeller), and these sound sources include frequencies that change with speed. The underwater signal, depending on the speed of the ship, will increase or decrease in direct proportion to the speed of the ship, which all have a linear relationship. Therefore, the analysis of a few main components will allow the calculation of the remaining parameters without much complexity.

The noise from a moving ship is mainly caused by propeller blades, which in turn causes cavitation. The International Institute of Marine Surveying in United Kingdom demonstrated that, according to Bernoulli’s Law, the propeller blade passing through the water exerts positive pressure on the blade’s surface and negative pressure on the blade’s back. Negative pressure leads to water bubbles around the blade, and when these bubbles explode, shock waves are created. The repetition of such processes produces ship-specific features. Based on that phenomena, DEMON-type algorithms will extract the lower modulating frequencies from the higher frequency noise.

### 2.1. Drawbacks of the DEMON Algorithm

The DEMON technique is a set of algorithms used to analyze narrow-band underwater signals based on the principle of treating the noise or the signals generated by propellers as the envelope of amplitude modulation of carrier waveform that has a specific frequency (such as small propellers or diving system pressure regulators). That envelope is specific for the cavitation noise and modulation waveform, which determines the periodicity of the propeller rotation with the fundamental frequencies.

In the basic DEMON algorithm as defined by Nielsen, x(t) is the acoustic signal that contains the noise of the propeller and the environment, presented by:

(1) x(t)=s(t)+n(t),

(2) s(t)=m(f,t)w(t),

where s(t) is a broadband signal formed by the modulation of a carrier waveform w(t) by a modulating waveform m(f,t), and n(t) is environmental noise. The modulating waveform m(f,t) is periodic with frequency *f*, thus $m^{2}\left(f,t\right)$ is also periodic, which can be expressed under a cosine formula [22] as follows:

(3) $$m^{2}\left(f,t\right)=\sum_{l=0}^{L}A_{l}cos\left(lcft+l\phi \right),$$

where $c=2\pi /f_{s}$, $f_{s}$ is the sampling frequency, $A_{l}$ is the expansion coefficient of $m^{2}\left(t\right)$, ϕ is the phase, and *L* is the number of coefficients. Because the square makes the left side of Equation (3) always positive, the coefficient must be selected to make the right side also positive.

**Figure 1:**
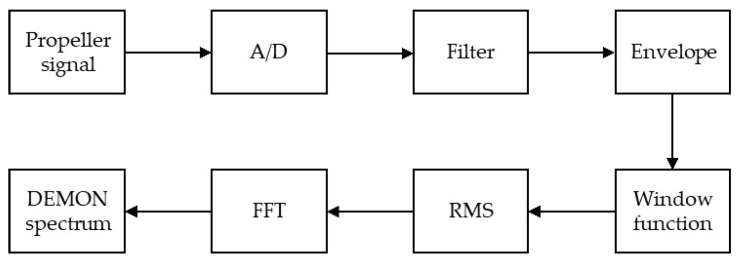
The orginal DEMON algorithm [4].

The Figure 1 shows the procedure to perform the DEMON algorithm. In this procedure, the propeller signal is first of all converted from analog to the digital signal. Afterwards, a bandpass filter is used to extract the designed cavitation noise signal. The envelope of the cavitation noise signal is then computed. A window function is used to capture a period of the envelop of the cavitation noise signal. After computing the root mean square (RMS) of the above-mentioned windowed signal, the Fast Fourier Transform (FFT) is applied to obtain the fundamental frequencies of cavitation noise, which are the typical features for detection and classification of the propellers.

DEMON was very effective at recognizing the diesel engine ships or propulsion steam boats; however, it began to expose limitations for new engine generations. The famous engine company Schottel, in 2015, introduced new versions of its advanced propulsion systems, such as new types of propellers and Schottel Rim Thruster (SRT) propulsion. The new SRT is an electric propulsion system with no gearbox or drive shaft. The static part of the electric motor is attached outside of the pipe connection. The propeller blades are attached inside of the rotating compartment. This creates a lighter propulsion device that reduces transmission loss and limits engine noise at very low levels. Therefore, it significantly affects the detection performance of the DEMON algorithm that focuses on processing the envelope frequency of the cavitation noise.

### 2.2. Improving the DEMON by Using Amplitude Variation

The signals emitted by the ship can be characterized by narrow-band frequency with low variations, because the motion of the ship engine and the propeller is normally stable. The spectrum of the received signals is characterized by the frequency peaks that exist in each period.

For the standard Fourier transform, the basic function is the complex oscillation:

(4) $$b_{w}\left(t\right):=exp\left(iwt\right),$$

where *t* is the time axis of the signal and *w* is the single frequency parameter. The Fourier transform of the signal s(t) is then written as an integral:

(5) $$F\left(w\right)=\int_{-\infty }^{\infty }exp\left(iw\tau \right)s\left(\tau \right)d\tau .$$

FFT cannot identify the time when the frequency occurs and the sound sources that the signal belongs to. Therefore, we used the Short Time Fourier Transform (STFT) instead of the Fast Fourier transform (FFT). The STFT adds a time dimension to the base function parameters by multiplying the infinitely long complex exponential with a window:

(6) $$b_{\left(w,t_{0}\right)}\left(t\right):=w\left(t-t_{0}\right)exp\left(iwt\right),$$

where w(t) is the window function and $\left(w,t_{0}\right)$ is the time-frequency coordinates of the base function. The general formula for STFT [23] is written as follows:

(7) $$S\left(w,t_{0}\right)=\int_{-\infty }^{\infty }w\left(t_{0}-\tau \right)exp\left(iw\tau \right)s\left(\tau \right)d\tau .$$

The formula shows information on the time and frequency domain. The result of Formula (7) is like using a filter-bank with band-pass filters, which have the Fourier transform as the window w(t) as the frequency response, but shifted to the center frequency *w*. STFT performs a series of FFT operations on each overlapping segment over the entire data.

Optimizing the STFT first usually involves finding the segment size, then adding zero-padding for small segment sizes to gain spectral better, and lastly, choosing an appropriate window.

**Figure 2:**
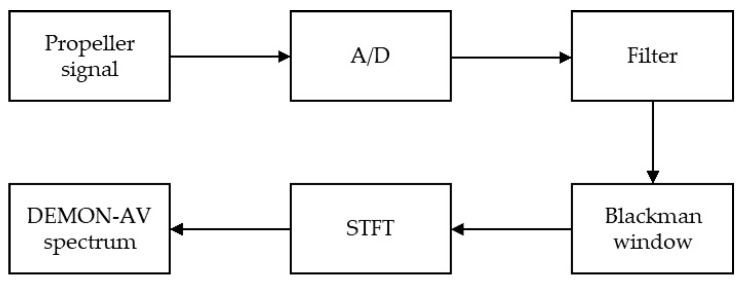
The proposed DEMON-amplitude variation algorithm.

We propose the DEMON by using an amplitude variation (the DEMON-AV), illustrated in Figure 2, as follows:

Step 1 The signal spectrum is calculated by STFT. From that, we calculate a two-dimensional spectral matrix, among which one dimension is the frequency, and the other is the number of samples.
Step 2 The algorithm considers the frequency spectrum according to the dB scale of the signal as input. We divide the dataset into samples with an equal length of time. Underwater signals are complex by nature, which are influenced by many environmental factors. If selecting records with short lengths of time, there will not be enough characteristics for analysis, thus the analysis quality is reduced. Conversely, if selecting records with long lengths of time, it will be redundant and ineffective. Therefore, based on the actual calculations and the amount of available data, our research divided the data samples into 30 s long samples which proved to be optimized. Then, we computed the amplitude variation for each segment of the spectrum, and constructed a spectral matrix.
Step 3 The frequency amplitude of each segment is averaged to obtain a unique representative value.
Step 4 Using the stacking technique to reduce the variance to increase the signal-to-noise ratio (SNR) and the threshold of the signal-to-noise ratio was selected based on calculating the standard deviation of the original signal spectrum.

This technique divides the acoustic signal x(t) into consecutive overlapping segments. The variance will decrease as we divide more overlapping segments; thus, the probability of correctly estimating the fundamental frequency *f* of the signal will increase.

### 2.3. Simulation Results and Evaluation of Detection Accuracy

**Figure 3:**
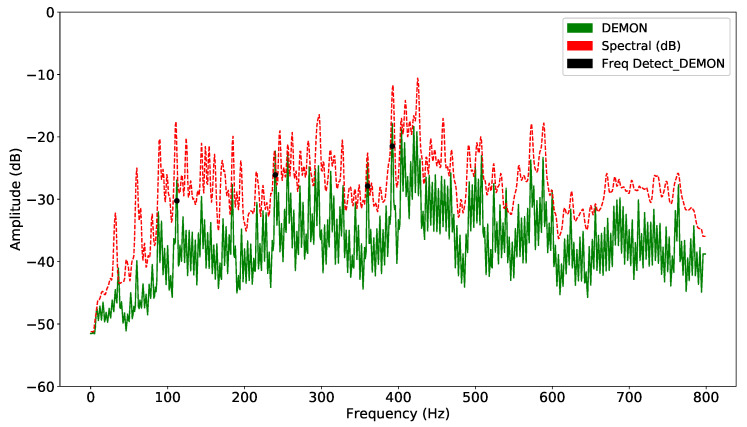
Signal frequency detection by DEMON with Record-1.

**Figure 4:**
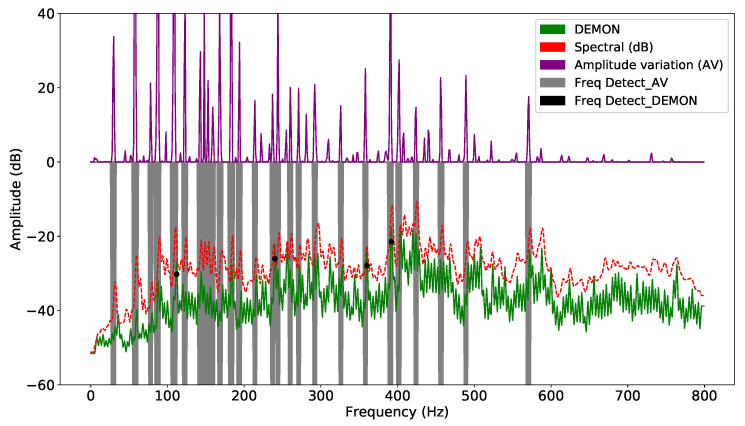
Signal frequency detection by DEMON-AV with Record-1.

**Figure 5:**
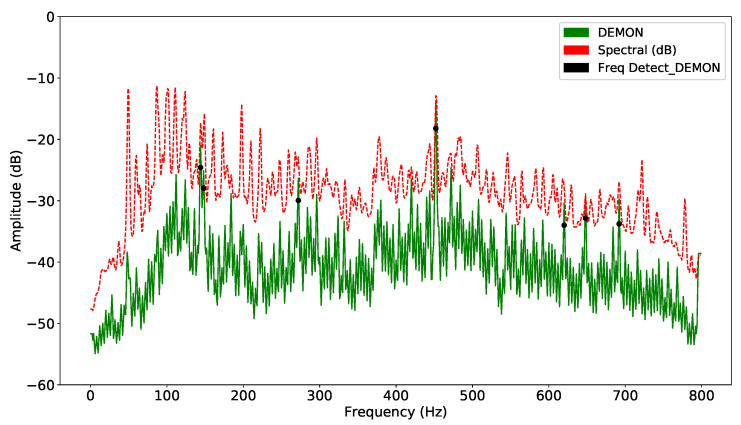
Signal frequency detection by DEMON with Record-2.

**Figure 6:**
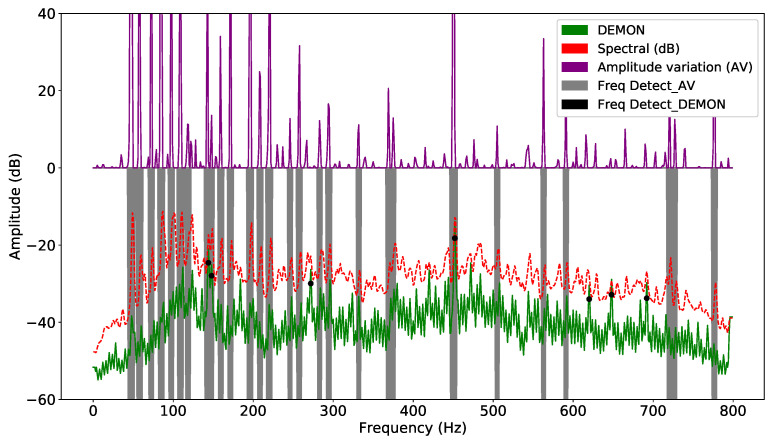
Signal frequency detection by DEMON-AV with Record-2.

In both methods, computation requires the definition of a target frequency window; unsuitable selection of input parameters can make the detection task unfeasible. Each sample is smoothed by the window function, and the corresponding standard deviations are calculated. The signal is detected whenever the corresponding signal exceeds the corresponding detection threshold. The frequency amplitude of each segment is averaged to obtain a unique representative value. This technique divides the acoustic signal into consecutive overlapping segments. When the signal is unstable, the detection and classification accuracy will be reduced significantly. DL models can solve this problem more easily, because they extract hidden features using layers. On the other hand, as there are various types of noise, suitable selection of features plays an important role in guaranteeing the performance of the model. Thus, the result of improving DEMON by amplitude variation will reduce false alarms, while retaining sufficient features to increase detection accuracy. DEMON considers the adjacent frequencies to be an envelope, therefore, it can only detect one maximum peak frequency. On the other hand, DEMON-AV focuses on calculating the amplitude variation between adjacent frequencies, then by observing the frequency changes along the signal spectrum, it can show the characteristics of each amplitude peak frequency. Based on the recorded data (Record-1), the Figure 3 shows the signal frequencies detected by the original DEMON algorithm, whereas the Figure 4 illustrates the detected results by using the DEMON-AV. It is clear that the proposed DEMON-AV shows a better performance in terms of characteristic frequencies and harmonics separation for a given measured acoustic data, when it is compared to the original DEMON. Based on the Record-2, the similar results can be obtained by using the original DEMON and the DEMON-AV, which are plotted in the Figure 5 and the Figure 6, respectively.

Detection accuracy is calculated from the numbers and percentages of correct and incorrect ship detection. We selected 3300 samples to test the analysis results, among which 1800 samples contained signals from the subjects, and 1500 samples did not. Each sample was divided into equal lengths of a minute. Table 1 shows two confusion matrices displaying the detection accuracy, and Table 2 summarizes the accuracy rates.

**Figure 7:**
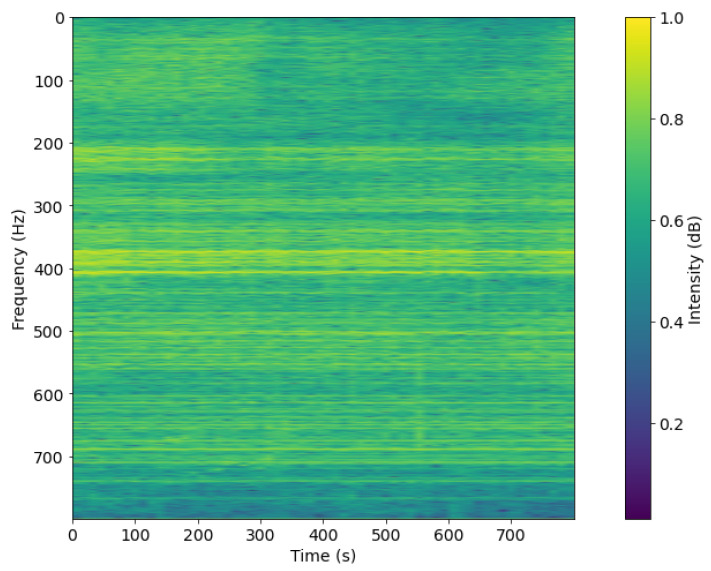
Spectrogram after pre-processing with DEMON-AV for Record-1.

**Figure 8:**
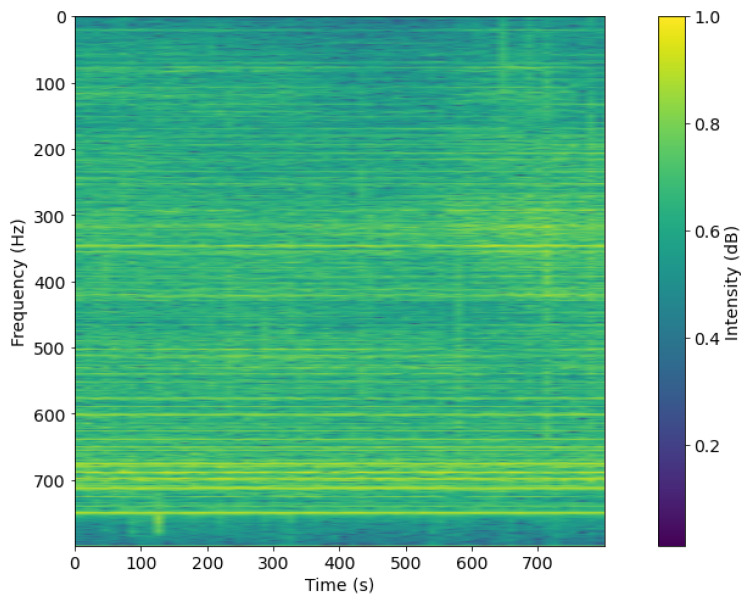
Spectrogram after pre-processing with DEMON-AV for Record-2.

The result of this process, such as Figure 7 and Figure 8, is a set of filtered spectrogram images, which will be put into the DL network for training.

## 3. Using CNN for Spectrogram Classification

Passive sonar generally relies solely on the ability of surveyors to directly hear underwater signals or look at the spectrum. However, human error can cause different results at different times on the same signal. Applying the neural network to a passive sonar is a form of support that adds references for the surveyors, helping the system to operate more stably.

### 3.1. Reasons for Selecting CNN

A person with normal vision can always detect and recognize objects in an image, as well as describe the content. However, this task is much more difficult for a computer, as it considers each image to be merely a numerical matrix (a set of pixels represented numerically in a specific system, usually Red-Green-Blue (RGB). Therefore, we need to find a bridge that connects this numerical matrix with the semantic information contained in the image. ANN does not work very well with image input. In the data set processed in Section 2, if we consider each pixel as a feature, an input image with dimensions (224 × 224 × 3) will have 150, 528 features. If the image size increases to 1000 × 1000, each input image will have 1 million (1 M) features. If using fully connected NN and assuming the second layer has 1000 units, the matrix size will be 1000 × 1 M, which equals to a weight of 1 billion (1 B) to be trained. This requires a huge amount of computations and often leads to overfitting due to insufficient training data. Therefore, our research team proposed to use proposed CNN to extract and classify underwater signals based on the spectrogram image data processed in Section 2.

### 3.2. CNN Architecture and Proposed CNN

LeNet was one of the first CNN models developed by Yann LeCun. LeNet’s structure consists of two convolution layers, two maxpooling layers, two fully connected layers, and the output is a softmax layer. The downsides of LeNet are that the network is very simple, and it uses sigmoid (or tanh) functions in each convolution layer, so the network computes very slowly.

VGGNet uses a series of convolution layers in the middle and the end of the architecture. This will make the whole computation process take longer, but the features will be retained better than using maxpooling. Input size decreases through convolution, but the depth increases. Due to the depth and the number of fully connected nodes, the VGG16 network and variants, such as VGG19, have enormous capacities. This makes implementing VGG a difficult task.

Like other hidden layers, the convolution layer takes input data and transforms this to produce input for the next layer (the output of this layer is the input of the next layer). The transformation used is the convolution calculation. Each convolution layer contains one or more filters which are feature detectors that detect and extract different features of spectrograms. The general formula of a continuous domain one-dimensional convolution is defined by:

(8) $$\left(f*g\right)\left(t\right)≜\int_{-\infty }^{\infty }f\left(\tau \right)g\left(t-\tau \right)d\tau .$$

In CNN, f(t) is the input image, g(t) is the filter that acts as a sliding window, and *t* is the position of the filter placed on the original spectrogram. At each point *t*, (f∗g)(t) is the value of the product of the intersection between the signal and the window with the delay *t*. That integral gives the relative correlation between the signal and the window in the defined domain. Moving from a continuous to a discrete domain by using the Riemann sum formula yields:

(9) $$\left(f*g\right)\left(t\right)≜\sum_{\tau \in D_{f}\vee t-\tau \in D_{g}}f\left(\tau \right)g\left(t-\tau \right).$$

The Riemann sum formula estimates an integral by dividing the domain into intervals and calculating the area of each interval.

**Figure 9:**
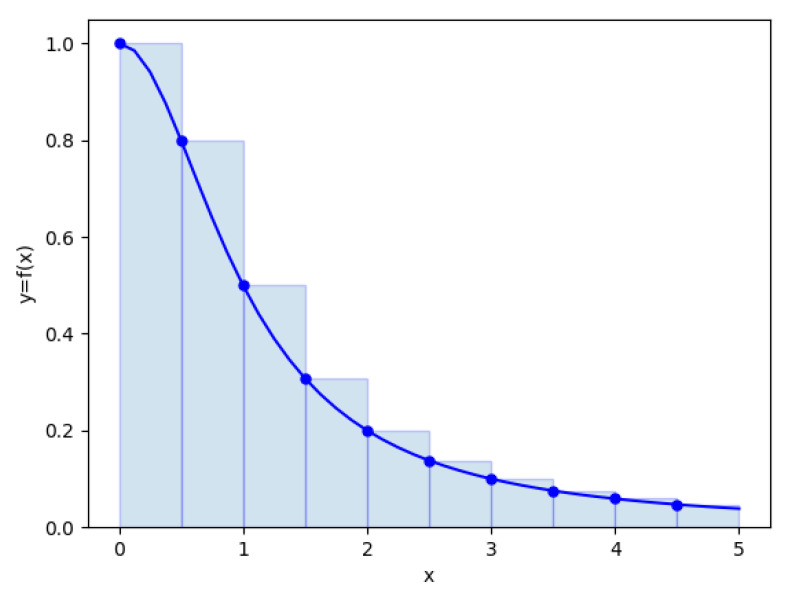
Examples of the Reimann sum.

The number of intervals above is the size of the filter in the convolution layer. If the filter size is [5 × 5], we multiply the convolution 25 times and then add them up, instead of integrating the whole region continuously. In Figure 9, the example of the Riemann sum, instead of having to calculate the function value at all the points from 0 to 5, we only need to calculate the results of 10 intervals with a 0.5 difference, thereby decreasing the number of calculations from infinity to 10. The calculated points are called the “stride”. We will use the stride to move the filter with each specified step. The smaller the stride, the more we have to compute, and as a result, the size of the output gets bigger. The larger the stride, the less we have to compute, but we will lose more relevant information.

**Figure 10:**
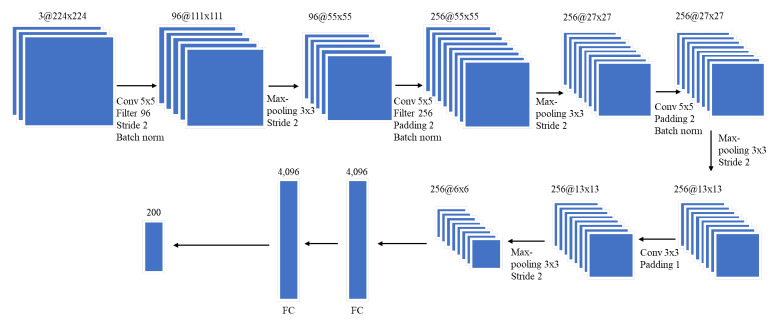
Proposed CNN structure.

The proposed network model structure diagram is shown in Figure 10:

Pixels at the center of the input matrix are covered by sliding a window over and over, meaning that the area is used many times to calculate the output value; whereas the pixels at the corners or edges of the matrix are only used a few times, so a lot of important information in the near-edge areas will be lost. However, this is the area that contains a lot of frequency information in the spectrogram. Therefore, we added padding with a value of 2, meaning that the input will be buffered with zero values, so that the model can do the integration. Without padding, the window will only slide where the window and the signal completely intersect. Adding padding increases the size of the input matrix, leading to an increase in the output matrix size. Therefore, the positions on the edges and corners of the original input matrix recede deeper, which will be used more in calculating the output matrix, avoiding information loss.

The tuning is a challenge with a deep-learning complicated structure. Because underwater datasets are insufficient, it is difficult for the deep model network to be trained. Therefore, we propose a neural network using batch normalization using one input layer, four convolution layers, four maxpooling layers, and two fully connected layers. The batch normalization layers which are placed just after defining the sequential model and after the convolution layer will reduce the internal covariate shift of the model. The internal covariate shift is a change in the input distribution of an internal. The inputs received from the previous layer are always changed. Adding batch normalization layers ensures that the mean and standard deviation of the inputs will always remain the same, and minimize the fluctuation of the distribution. Batch normalization is placed after the activation layers, because if we put batch normalization beforehand, then the calculation of batch normalization will likely produce negative features, and applying activation layers like Relu will lead to the loss of image characteristics. Batch norms do not compute the entire data, and the model’s data distribution will make some noise. This can help overcome overfitting and help learn better. The first convolution layer has one convolution [5 × 5], and the stride is two, with 96 kernels. Using a smaller convolution matrix [5 × 5] will retain more information on the spectrogram. If the matrix size is an even number, we have to add padding to the left of the input matrix more than the right (or vice versa), which results in an asymmetry. Therefore, we chose the matrix size as an odd number, in order to have a pixel in the center. This can be regarded as a distinguishing point for better performance. The filter size of the pooling layers is [3 × 3]; the stride is two. Extending the size of the convolution layers, reducing the dimensions of the feature map and making the filter size and stride smaller increased the accuracy of our model.

### 3.3. Training Model with Pre-Processing Data

**Figure 11:**
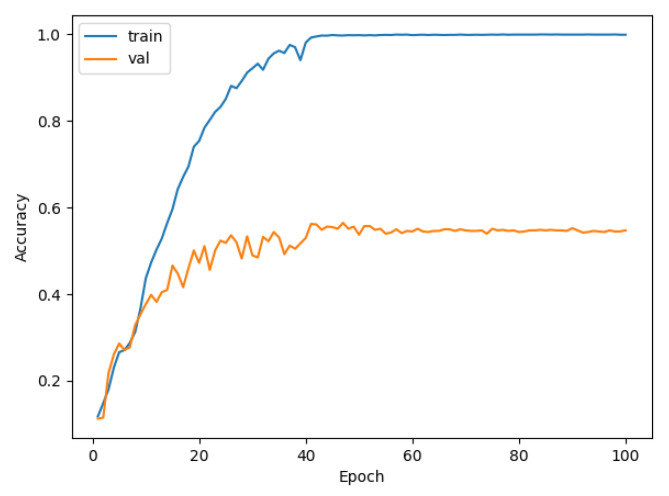
The accuracy of DEMON and LeNet (54%).

**Figure 12:**
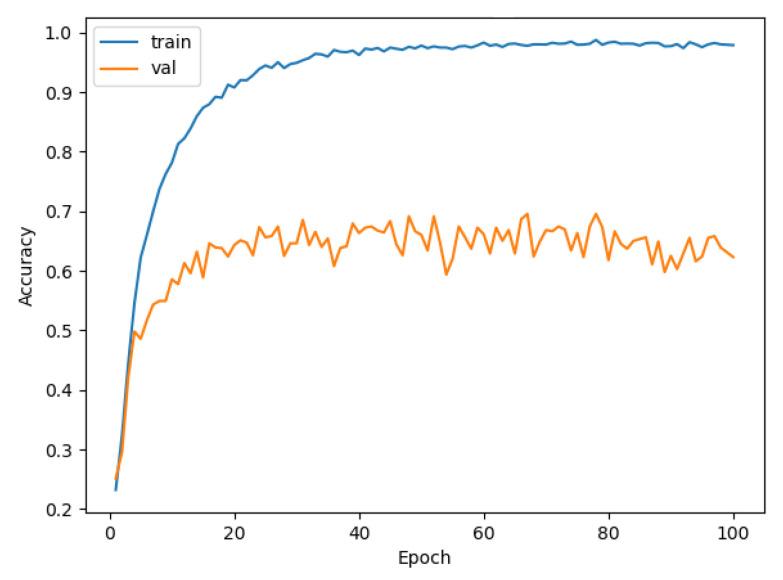
The accuracy of DEMON and VGG (63%).

**Figure 13:**
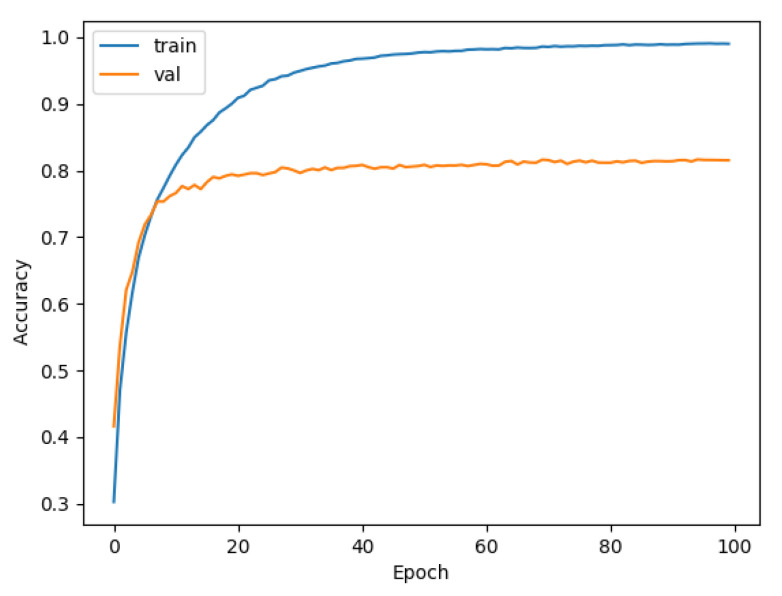
The accuracy of DEMON and proposed CNN (80%).

We used data sets that had been pre-processed by DEMON and DEMON-AV as inputs to LeNet, VGG, and our proposed CNN model. It is easy to see the difference in the accuracy between the models. We separated the samples into 70% for the training set, 20% for the validation set, and 10% for the test set. We also used a spectrogram size of (3 × 224 × 224) to include in the CNN model for training. From analyzing results between the models, the accuracy of LeNet and VGG with DEMON pre-processing is only 54%, as shown in Figure 11, and 63%, as depicted in Figure 12, respectively. The training time of LeNet is 3.5 h, while that of VGG is 4 h. Our proposed CNN pre-processed by DEMON reached the accuracy level of 80% as illustrated in Figure 13, which is higher than the two previous models, with the same training time.

**Figure 14:**
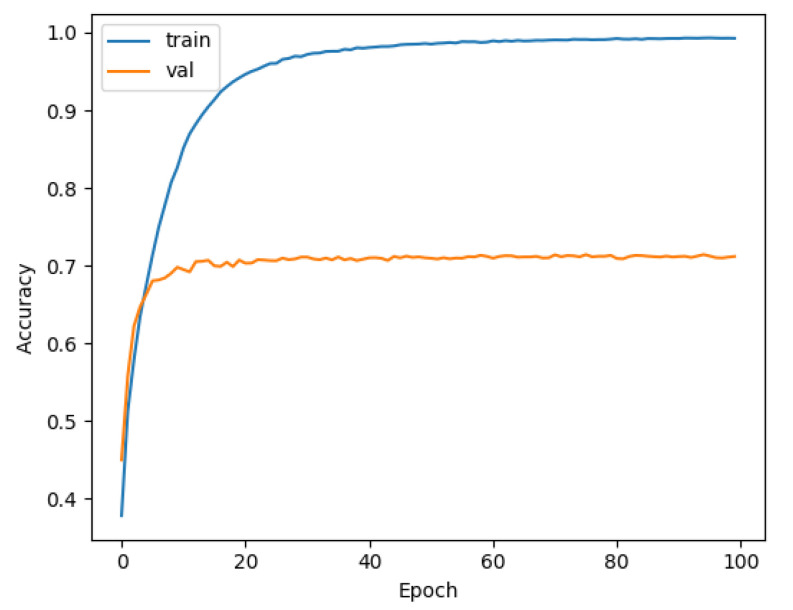
The accuracy of DEMON-AV and LENET (70%).

**Figure 15:**
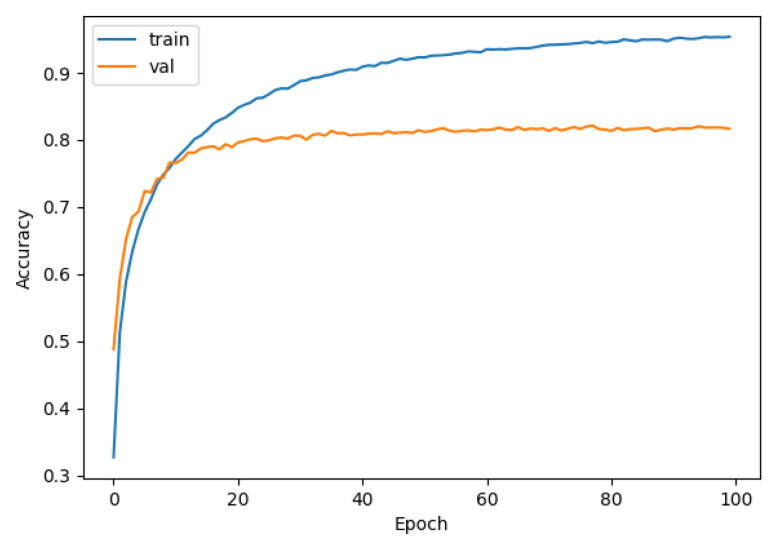
The accuracy of DEMON-AV and VGG (78%).

**Figure 16:**
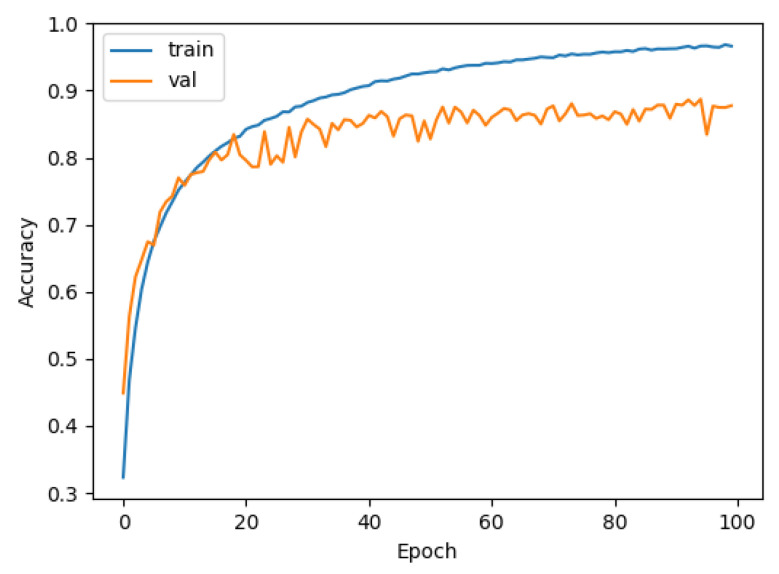
The accuracy of DEMON-AV and proposed CNN (90%).

Similarly, we tested LeNet and VGG using DEMON-AV pre-processing for a training time of 4 h, and obtained the accuracy result of 70%, as shown in Figure 14, and of 78%, as given in Figure 15, respectively. Finally, we pre-processed our proposed CNN with DEMON-AV, and the obtained accuracy result is 90% as depicted in Figure 16, which is the highest accuracy obtained by all tested models.

We can conclude that our proposed model has significantly improved the accuracy without increasing the training time. It proves that improving the traditional signal processing combined with improving the DL model will remarkably increase classification performance. In DL, a model can be viewed as a “black box”, in which operations between the input and output of the system are not visible to viewers. The model will be optimized for each specific purpose to focus on each desired outcome. Thus, it is not only the security of the system, but also the desired accuracy which is enhanced.

**Figure 17:**
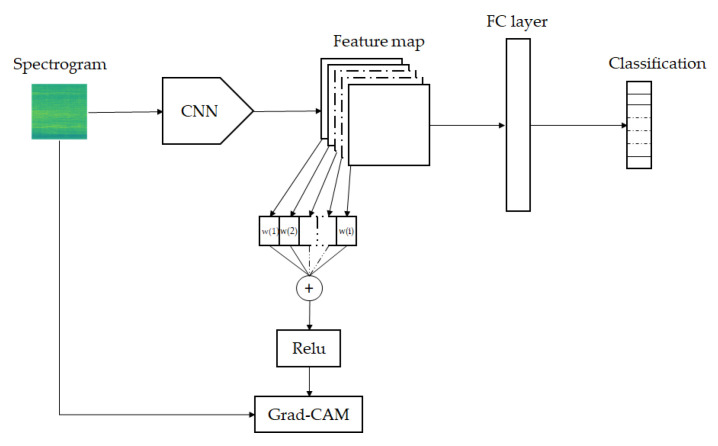
Grad-CAM flow chart [24].

To obtain the heat map, we used Gradient-weighted Class Activation Mapping (Grad-CAM) [24], as demonstrated in Figure 17, to compute the gradient of the score for a specific feature of a convolution layer. Those gradients were global-average-pooled to obtain the total weights $\alpha_{c}^{k}$ with c, which is the class:

(10) $$L_{Grad-CAM}^{c}=ReLU\left(\sum_{k}\alpha_{k}^{c}A^{k}\right).$$

**Figure 18:**
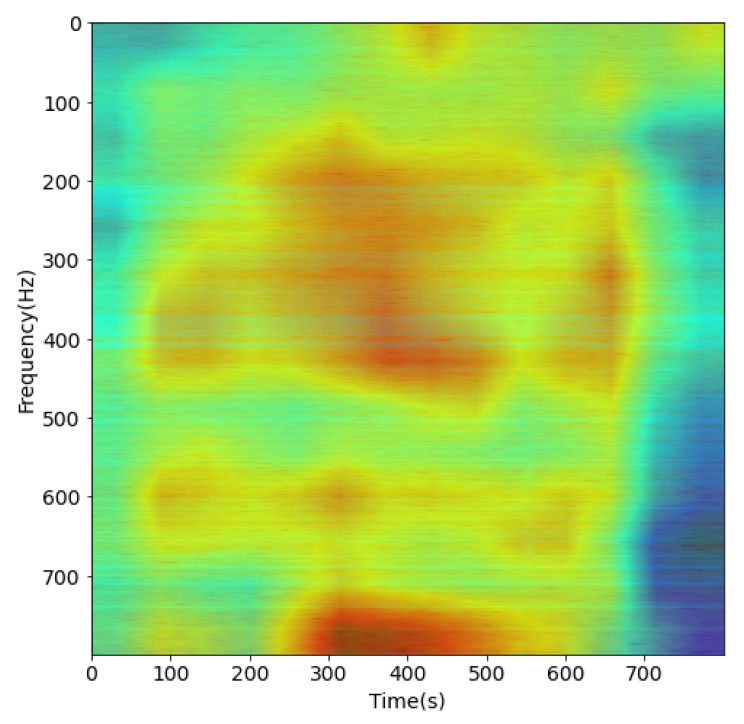
Proposed CNN Heatmap with spectrogram of Record 1.

**Figure 19:**
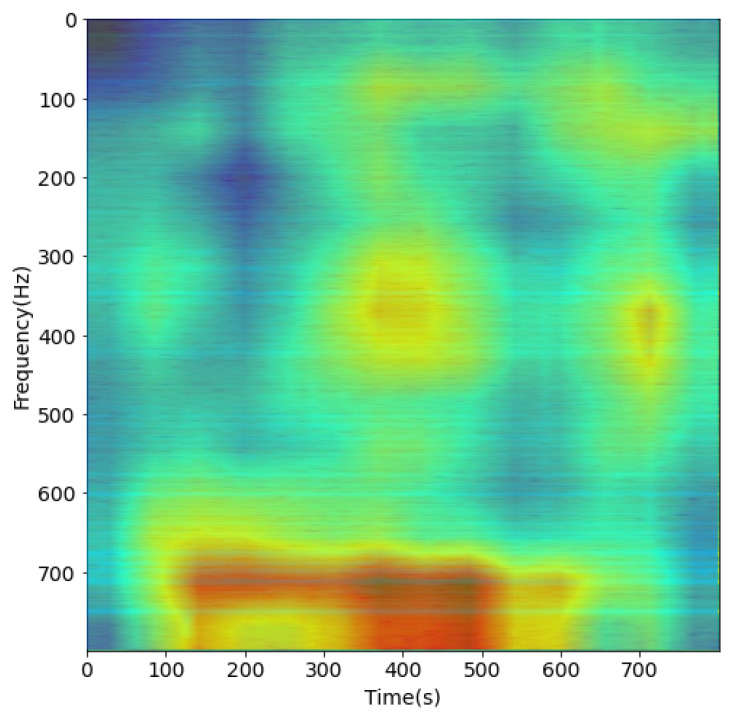
Proposed CNN Heatmap with spectrogram of Record 2.

The Figure 17 shows the Grad-CAM flow chart proposed in [24]. We used Relu activation to combine all weighted feature maps to visual Grad-CAM heatmaps as depicted in Figure 18 and Figure 19.

We checked the transparency of the proposed CNN model with a heatmap to visually explain whether the key input regions containing the features from each spectrogram were correctly and sufficiently extracted or not. The high-resolution visualization defines the important features and class discrimination.

From the heat-maps in Figure 18 and Figure 19, we found that the proposed CNN could precisely extract the area that contains the feature of the input signal. All training and testing in this paper were conducted on Python 3.6.8 in the Ubuntu 20.04 system. The environment used to configure the GPU was CUDA 10.1 and Cudnn 7.6.5 on Dell T3600 Workstation Xeon 8 core NVIDA k2200 4 GB.

## 4. Experimental Results

In fact, when researching the characteristics of the underwater environment as well as sonar systems, there are many different channels to model and systems for receiving and analyzing signals. The research team’s basic system uses a single channel, consisting of a hydrophone, a module for signal processing, and signal amplification modules (digitizer and filter) and a real-time recorder. The system can expand to measure multiple channels by increasing hydrophones for each specific case.

**Figure 20:**
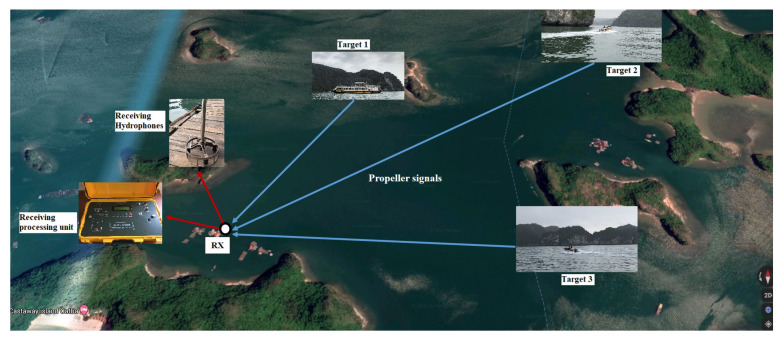
Location of receiver units and some targets on Google Maps.

Our simulation used the dataset from the project: “An underwater vessel noise data-base” by the Research Center for Telecommunication Technologies—Universida de Vigo [25], as well as the dataset recorded by ourselves—the Institute of Electronics—in Lan Ha Bay, Hai Phong, Vietnam. Datasets include various types of underwater ship sounds. The underwater channel used for data collection was built on the geometry channel which were introduced and demonstrated by Van Duc Nguyen et al. [26,27]. The sounds were recorded in shallow waters in real conditions at different times during the day, which contains both natural and anthropogenic environment noise. The system uses hydrophones BII-7001-SN:0602, a product of Benthoway in Canada. BII’s is a ommi-spherical hydrophone which can receive a low frequency until 10 Hz, and offer excellent acoustic characteristics of low noise and durability. Bespoke built-in pre-amplifiers allow the hydrophones to be used with long extension cables with no loss in sensitivity [28]. The scenario for experiments is shown in Figure 20.

## 5. Conclusions

This paper described a method for surface vehicle propeller cavitation noise (VPCN) in shallow water. It is a spectrogram domain analysis for a passive sonar using amplitude variation with a modified Convolution Neural Network which attains an accuracy level of around 90% without increasing the training time. The proposed model, which is provided for cavitation noise from the propeller, has better performance in recognizing and decreasing false alarms. Based on the classification results, we conclude that: (1) Deep learning models provide good results for detecting and classifying underwater and surface targets, and these models still process well in low-SNR environments; (2) while the DEMON algorithm focuses on fundamental frequency, our improved model additionally recognizes variations in the amplitude of fundamental frequencies; (3) the transformation of data from the signal sequence to the spectrogram enables the system to process a large amount of complicated data on a real-time basis; and (4) datasets are still limited due to some security reasons. Therefore, pre-processing datasets and finding ways to increase the number of samples are the two main problems that shall be improved in the future.

## Figures and Tables

**Table 1:** Detection accuracy on a database of 3300 samples.

**DEMON**	**Ship**	**No Ship**
Ship	1463	198
No Ship	337	1302
Total samples	1800	1500
**DEMON-AV**	**Ship**	**No Ship**
Ship	1768	45
No Ship	32	1455
Total samples	1800	1500

**Table 2:** Accuracy rates and also the false-alarm rates.

	DEMON (%)	DEMON-AV (%)
Detection Accuracy1	81.28%	98.22%
False Alarm	13.2%	3%

## Abbreviations

The following abbreviations are used in this manuscript:

ANN	ArtificialNeural Networks
CNN	Convolution Neural Network
DEMON	Detection Envelope Modulation On Noise
DEMON-AV	Detection Envelope Modulation On Noise-Amplitude variation
FFT	Fast fourier transform
Grad-CAM	Gradient-weighted Class Activation Mapping
RMS	Root mean square
STFT	Short time fourier transform
VPCN	Vehicle propeller cavitaion noise
